# Natal origin affects host preference and larval performance relationships in a tritrophic system

**DOI:** 10.1002/ece3.2826

**Published:** 2017-02-26

**Authors:** Thomas A. Verschut, Laima Blažytė‐Čereškienė, Violeta Apšegaitė, Raimondas Mozūraitis, Peter A. Hambäck

**Affiliations:** ^1^ Department of Ecology, Environment and Plant SciencesStockholm UniversityStockholmSweden; ^2^ Laboratory of Chemical and Behavioural EcologyInstitute of EcologyNature Research CentreVilniusLithuania

**Keywords:** *Asecodes lucens*, *Galerucella sagittariae*, larval performance, natal experience, oviposition preference, tritrophic interactions

## Abstract

Many insects face the challenge to select oviposition sites in heterogeneous environments where biotic and abiotic factors can change over time. One way to deal with this complexity is to use sensory experiences made during developmental stages to locate similar habitats or hosts in which larval development can be maximized. While various studies have investigated oviposition preference and larval performance relationships in insects, they have largely overlooked that sensory experiences made during the larval stage can affect such relationships. We addressed this issue by determining the role of natal experience on oviposition preference and larval performance relationships in a tritrophic system consisting of *Galerucella sagittariae,* feeding on the two host plants *Potentilla palustris* and *Lysimachia thyrsiflora,* and its larval parasitoid *Asecodes lucens*. We firstly determined whether differences in host‐derived olfactory information could lead to divergent host selection, and secondly, whether host preference could result in higher larval performance based on the natal origin of the insects. Our results showed that the natal origin and the quality of the current host are both important aspects in oviposition preference and larval performance relationships. While we found a positive relationship between preference and performance for natal *Lysimachia* beetles, natal *Potentilla* larvae showed no such relationship and developed better on *L. thyrsiflora*. Additionally, the host selection by the parasitoid was mainly affected by the natal origin, while its performance was higher on *Lysimachia* larvae. With this study, we showed that the relationship between oviposition preference and larval performance depends on the interplay between the natal origin of the female and the quality of the current host. However, without incorporating the full tritrophic context of these interactions, their implication in insect fitness and potential adaptation cannot be fully understood.

## 1 Introduction

Insects are faced with the challenge to find egg‐laying sites where the environmental (Fritz, Crabb, & Hochwender, 2000; Mayhew, 1997; Mitchell, 1981) and biotic conditions (Hilker & Meiners, 2011; Randlkofer, Obermaier, Hilker, & Meiners, 2010; Thompson, 1988) are most suitable for their offspring. Due to the problem of host finding in complex environments, both herbivorous insects and their natural enemies use sensory experiences made during the larval stage as a sensory shortcut to locate suitable hosts (van Emden et al., 1996; Gripenberg, Mayhew, Parnell, & Roslin, 2010; Sutter & Kawecki, 2009). Furthermore, in some insect species, larval experiences can cause physiological adaptations to the diet of their natal habitat (Cornell & Hawkins, 2003; Ehrlich & Raven, 1964; Stamps, Luttbeg, & Krishnan, 2009). These natal experiences may bias insects to use similar hosts across generations and have been suggested to lead to intraspecific variation in habitat and host choice (Davis, 2008; Davis & Stamps, 2004; Immelmann, 1975), which eventually can lead to host race formation (Pappers, van der Velde, & Ouborg, 2002; Pfennig et al., 2010; Via, 1999), or sympatric speciation (Beltman, Haccou, & ten Cate, 2004; Bernays, 2001).

Similar to herbivorous insects, female predators and parasitoids need to select resources based on larval performance in order to maximize their own fitness. For instance, many studies have shown that the offspring size and survival for larval parasitoids increase with the size and growth rate of host larvae (Fournet, Poinsot, Brunel, Nenon, & Cortesero, 2001; Godfray, 1994; Harvey, van Dam, & Gols, 2003; Stenberg & Hambäck, 2010). Moreover, female parasitoids allocate more female than male offspring to larger hosts (Vet, Datema, Janssen, & Snellen, 1994; Zaviezo & Mills, 2000), as female offspring is more valuable in terms of reproductive output (Bernal, Luck, & Morse, 1998; Charnov, Los‐den Hartogh, Jones, & van den Assem, 1981; Godfray, 1994). Thus, when the selection by herbivore females aims to maximize larval growth on a given host plant, this selection may also increase the fitness of the parasitoids attacking these larvae (Bernays, 1988; Godfray, 1994; Price et al., 1980). Especially for parasitoids, it has been found that using chemical information of the natal habitat to differentiate among host resources can have strong consequence on offspring fitness (Dukas & Duan, 2000; Harvey, Gols, Snaas, Malcicka, & Visser, 2015; van Nouhuys, Reudler, Biere, & Harvey, 2012). For example, the work of van Emden et al. (1996) suggested a chemical legacy where chemicals on the surface of their aphid host mummy affected the host selection of the parasitoid female. Not surprisingly, herbivores and their natural enemies frequently use similar chemical information to locate suitable host plants and suitable host insects (Fatouros et al., 2012; Takabayashi et al., 1998; Vet & Dicke, 1992), causing some herbivorous insects to select host plants that are suboptimal for larval growth and survival (Ballabeni, Wlodarczyk, & Rahier, 2001; Bernays, 1988; Denno, Larsson, & Olmstead, 1990).

The potential correlation in host use across trophic levels suggests that the influence of natal experience should not be studied in separate pairwise interactions between host plants and herbivorous insects (Gripenberg et al., 2010; Singer, 1983), or between host insects and their parasitoids (Harvey et al., 2015; Price et al., 1980), but in its full tritrophic context. While a few studies have explored the effects of oviposition preference and larval performance of herbivorous insects and their natural enemies in the same system (Harvey et al., 2015; Mooney, Pratt, & Singer, 2012), the effect of natal experience has largely been neglected. In this study, we aim to bridge this gap, by including the role of natal experience in a study on oviposition preference and larval performance relationships in a tritrophic system. As a study system, we used the oligophagous herbivorous leaf beetle *Galerucella sagittariae* Gyllenhaal (Coleoptera: Chrysomelidae), its larval parasitoid *Asecodes lucens* Nees (Hymenoptera: Eulophidae), and two co‐occurring but unrelated host plants of the beetle, *Potentilla palustris* (L.) Scop. (Rosaceae) and *Lysimachia thyrsiflora* (L.) (Primulaceae).

**Figure 1:**
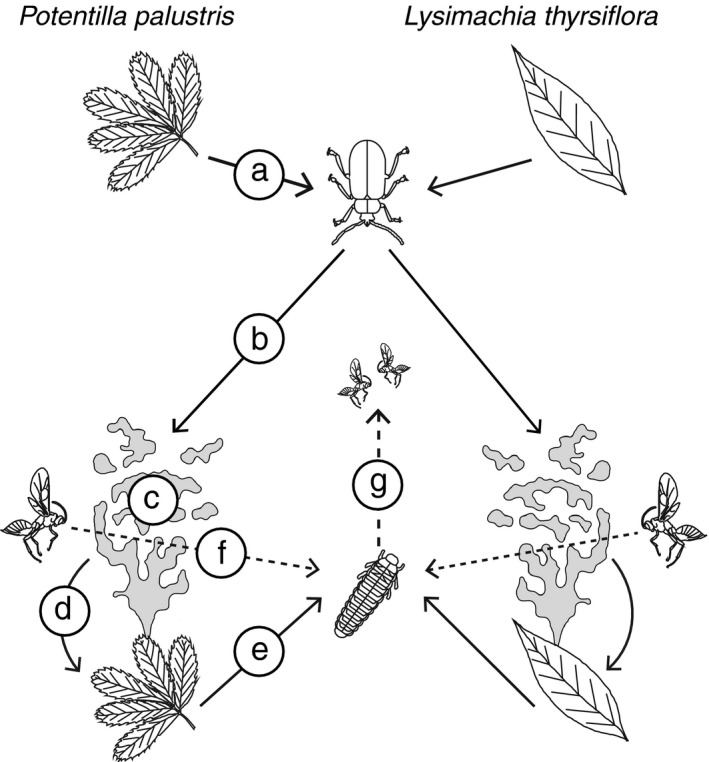
Conceptual diagram illustrating the tritrophic interaction. For both *Galerucella sagittariae* (solid lines) and *Asecodes lucens* (dashed lines), we collected adults originating from *Potentilla palustris* and *Lysimachia thyrsiflora* (a—section 2.2). The behavioral components that were tested are as follows: (b—section 2.3) the olfactory preferences of *G*. *sagittariae* for both host plants; (c—section 2.4) antennal responses of *G*. *sagittariae* to headspace volatiles (gray odor plumes) of both host plants; (d—section 2.5) the oviposition preference of *G*. *sagittariae* on both host plants*;* (e—section 2.6) the performance of *G*. *sagittariae* larvae on both host plant species; (f—section 2.7) the olfactory preferences of *A*. *lucens* to larval‐ and host plant‐derived volatiles; (g—section 2.8) the performance of *A*. *lucens* on host larvae from both host plants. For both the preference and performance relationship of *G*. *sagittariae* and *A*. *lucens*, we performed a full factorial design in which both natal origin and current host were used as factors, but for simplicity we did not include all interactions in the diagram

We used a full factorial design in which both natal origin and current host were included as factors to explore preference and performance relationships in the tritrophic system (Figure 1a). We firstly used laboratory experiments in which *G. sagittariae* of the two natal origins were allowed to select their preferred hosts exclusively on olfactory information (Figure 1b). In order to better understand the potential cues underlying the selective behavior, we combined these experiments with a quantification of volatile compounds and electrophysiological responses (Figure 1c). Subsequently, we conducted an oviposition experiment where adult *G. sagittariae* could also use other sensory cues for host selection (Figure 1d), and quantified the growth rate and final pupae size of the beetle larvae when feeding on the two host plants (Figure 1e). Finally, we tested whether *A. lucens* of both natal origins were able to select their preferred hosts exclusively based on olfactory information (Figure 1f), and determined whether the combination of host larvae and either of the two host plants affects the development of *A. lucens* offspring (Figure 1g). Using this setup, we thus determined the influence of natal origin on oviposition preference and larval performance relationships in tritrophic interactions.

## 2 Material and Methods

### 2.1 Study system

*Galerucella sagittariae* (formerly *G. nymphaeae* Linnaeus) is commonly found along lake shores, marshy riversides and wetlands, where it feeds on various Rosaceae, Primulaceae and Polygonaceae species (Hippa & Koponen, 1976; Nokkala & Nokkala, 1998). In our study area, *G. sagittariae* is mainly found on the two distinctive host plants *Potentilla palustris* and *Lysimachia thyrsiflora*. While *P. palustris* forms sprawling, vine‐like structured stems with pinnate leaves, *L. thyrsiflora* consists of an erect and unbranched stem with tapered leaves. In locations where both plants occur, *P. palustris* typically forms dense sprawling patches, whereas *L. thyrsiflora* often occur in small patches that are spatially separated from the *P. palustris* patches.

During late May, the overwintering *G. sagittariae* adults emerge from hibernation, and until the beginning of July, the adult beetles lay egg batches mainly on the lower side of the leaves. The eggs hatch after approximately 2 weeks and the larvae feed on the same plant for 3 weeks until they pupate on the lower side of the leaf. One of the main natural enemies of the larvae is the monophagous koinobiont parasitoid *Asecodes lucens*, which lay one to several eggs in second and third instar larvae. Unparasitized and parasitized larvae are easily separated because the parasitized larvae turn into a hardened black mummy in contrast to the normal beetle pupae (Dolgin, 1979). Within these mummified larvae, the parasitoids pupate and overwinter until late June of the following year, but for unknown reasons some adults already hatch in the same season (P.A. Hambäck & T.A. Verschut, personal observation).

### 2.2 Establishment of natal origins

We collected adult *G. sagittariae* beetles at a *P. palustris‐*dominated location (59°38′45.6″N, 18°10′03.3″E), and at a *L. thyrsiflora‐*dominated location (59°41′53.2″N, 17°55′06.2″E) in early spring. At both locations, the beetles are dispersed over relatively large spatial areas and while the other host plant is present in small numbers, they are seldom used as oviposition plant. The collected beetles were exclusively fed on the host plant of their origin and were used to establish two lines of beetles with distinct natal origins. Throughout this article, we refer to these beetles and their offspring as natal *Potentilla* beetles and natal *Lysimachia* beetles, respectively. Correspondingly, we collected late instar larvae at locations dominated by either of the two host plant species to acquire parasitoids of both natal origins. The late instar larvae were fed on their respective host plant until pupation. After pupation, the parasitized larvae were monitored for emerging parasitoids, and depending on the host plant from which the *G. sagittariae* larvae originated, we classified the offspring as either natal *Potentilla* or natal *Lysimachia* parasitoids.

To avoid that local adaptation of the insects to the host plants of their natal habitat would affect our result, we collected *P. palustris* (59°07′08.3″N, 17°17′13.0″E) and *L. thyrsiflora* (59°22′05.8″N, 18°03′58.6″E) from populations located approximately 100 km away from the locations where the insects were collected. All the collected host plants were potted in 1.5‐L peat filled pots and maintained in a controlled garden plot until they were used in one of the experiments.

### 2.3 Olfactory preferences of *Galerucella sagittariae*

We examined the ability of female beetles from both natal origins to discriminate between olfactory cues from the *P. palustris* and *L. thyrsiflora* in two‐armed olfactometers (Hambäck, Pettersson, & Ericson, 2003). The two‐armed olfactometers consisted of a central neutral zone (2.5 × 2.5 cm) and two tapered arm zones containing the test stimulus (3.8 cm long). At the beginning of each trial, an individual was allowed to acclimatize in the olfactometer for 5 min, after which the odors were introduced. We recorded the location of the beetles each minute for a total of 30 min, and we regarded the accumulated number of observations per test zone as an estimate of the time spent in the respective odor stimulus. All observations in the neutral zone, and individuals that moved less than five mm during five consecutive recordings were excluded from further data analysis.

**Figure 2:**
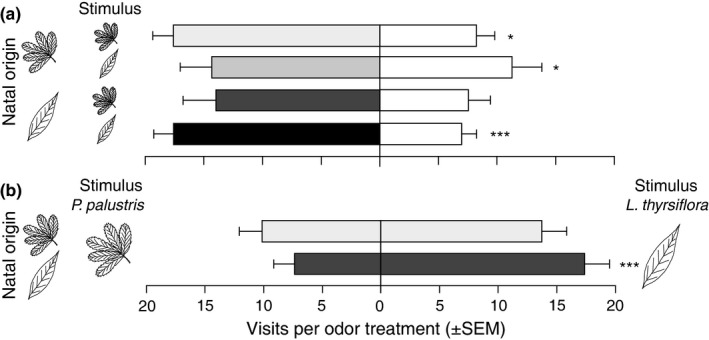
(a) Behavioral responses of natal *Potentilla* (*n* = 27 and *n* = 18, respectively) and natal *Lysimachia* beetles (*n* = 18 and *n* = 21) to odors from damaged *Potentilla palustris* and *Lysimachia thyrsiflora* (i.e., stimulus) vs. a control treatment with humidified air (white bars). Each shade of gray represents a specific combination of natal origin and current stimulus. (b) Behavioral response of natal *Potentilla* (*n* = 30) and natal *Lysimachia* beetles (*n* = 30) in two‐test odor experiments. Full overview of the data analysis is given in Table S1 (*p < .05; ***p < .001)

We firstly tested the olfactory response of beetles of both natal origins to olfactory cues from undamaged and damaged host plant vs. a control treatment of humidified air, which we denote as one‐test odor experiments throughout the rest of the paper. These tests indicated that beetles were only attracted to damaged plants, and we subsequently excluded undamaged plants from further analysis (Figure S1). We then compared beetle responses depending on natal origin when exposed to volatiles from feeding‐damaged plants of the two host species simultaneously, which we denote as two‐test odor experiments throughout the rest of the study (Figure 2). Feeding damage was obtained by allowing 10 conspecific adults to feed on plants for 20 hr in ventilated acrylic glass containers. Shortly before using the plants in the experiments, the beetles were removed from the plant. Depending on the treatment, shoots of 10–15 cm from either damaged or undamaged plants were placed in gas‐washing bottles that were connected to the olfactometer with Teflon tubing (ø 1.59 mm). The test odors were delivered into the olfactometer by pulling carbon filtered air through the gas‐washing bottles into the olfactometers at 6 ml/s (Model E flow meter, Kytola Instruments, Finland) with a diaphragm pump (MZ 2C; Vacuubrand GmbH, Germany). After each trial, the olfactometers were cleaned with a mild odorless detergent and ethanol, after which the positions of the odor treatments were switched.

### 2.4 Headspace collection and antennal response

To quantify differences in the volatile headspace of the damaged host plants, we collected volatile organic compounds (VOCs) and performed coupled gas chromatographic–electroantennographic detection (GC‐EAD) to determine which VOCs elicit antennal responses in *G. sagittariae* females (full methods available in Appendix S1). The VOCs were trapped in glass collection tubes filled with Tenax TA adsorbent (60/80 mesh; Sigma‐Aldrich AB, Sweden) through dynamic aeration in a push–pull system (Tholl et al., 2006). The odors collection tubes were extracted five times with intervals of 4 hr, after which they were combined and concentrated. The analyses were performed using a HP 6890N gas chromatograph (GC) equipped with a DB‐Wax capillary column (30 m × 0.25 mm × 0.25 μm; Agilent Technologies, USA) coupled to a HP 5973 mass spectrometer (MS; Agilent Technologies Inc, USA). After sampling, we removed all leaves from the stem and scanned the total leaf surface area and determined the damaged surface area (ImageJ v.1.48).

The GC‐EAD recordings were performed on reproductively mature females of both natal origins, which were exposed to VOCs of their respective host plant. The detached heads of the beetles were firstly connected to a silver wire grounded glass capillary electrode filled with 0.9% NaCl saline solution. Subsequently, the distal end of the antennae was connected to a recording electrode and the antennal signal was amplified and simultaneously recorded with the flame ionization detector (FID) signal (GC‐EAD V.4.4; Syntech, Germany). The GC‐EAD recordings were made on a Clarus 500 gas chromatograph (PerkinElmer, USA) equipped with a DB‐Wax capillary column in which a 1:1 effluent splitter allowed a simultaneous FID and EAD of the separated volatile compounds. All electroantennographically active compounds were identified by comparing their FID mass spectra with those in the NIST electronic MS library (v.2.0), then to published retention index values and finally to authentic standards using MSD Productivity ChemStation (v.02.01.1177). For all identified compounds, we determined the relative amounts of the compounds injected in the GC‐MS analysis as areas under the chromatographic peaks.

### 2.5 Oviposition preference of *Galerucella sagittariae*

**Figure 3:**
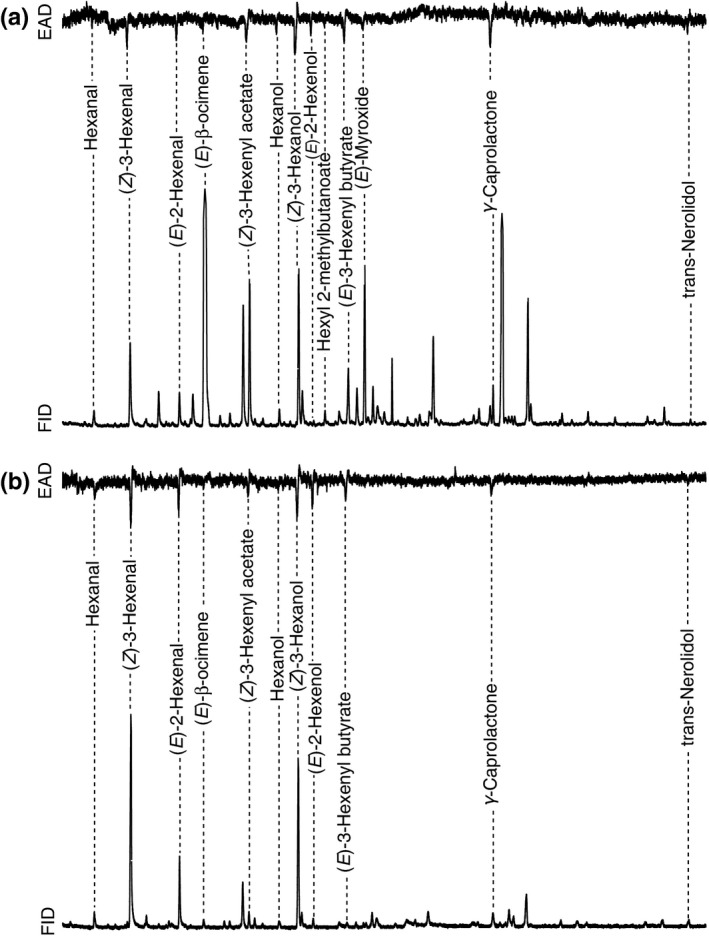
Averaged traces of the antennal response of (a) natal *Potentilla* (*n* = 5) and (b) natal *Lysimachia* (*n* = 4) females responding to volatile collections of their respective natal plant. The top trace represents the electroantennographic detection (EAD) of the *Galerucella sagittariae* females, and the lower trace represents the volatile compounds detected by flame ionization detection (FID) in (a) *Potentilla palustris* and (b) *Lysimachia thyrsiflora*. The dashed lines connect the peaks of the volatile compounds that caused a response in the antennae of the females of either natal origin. Summarized details of the GC‐EAD responses are shown in Table S2

The oviposition preference of *G. sagittariae* females of both natal origins for the two host plants, *P. palustris* and *L. thyrsiflora,* was tested in cage experiments during June and July 2015 in a controlled garden plot. Three males and three females, which had been separated for 3 days prior to the experiment, were placed in an outdoor cage (50 × 50 × 75 cm) covered with a fine mesh (0.4 mm) for 10 days. Each cage contained four host plants, with different frequencies of the two host plants (Figure 3). At the end of the experiment, the number of egg batches and the number of eggs per batch were counted to determine the oviposition preference.

### 2.6 Performance of *Galerucella sagittariae* larvae

To determine whether the natal origin and the oviposition choice of the female affect the performance of larvae on either host plant species, we measured growth rates under controlled conditions. We haphazardly collected leaves from both host plants containing egg batches laid during the oviposition experiment and stored them in Petri dishes with humidified filter paper until hatching. Within 12 hr upon hatching, we randomly selected 20 larvae per combination of natal and oviposition choice of the female (i.e., current host plant of larvae) and measured the initial weight on a microbalance (EP125 SM, Precisa Gravimetrics AG, Switzerland). Each larva was individually fed on leaves from the oviposition plant in a Petri dish with humidified filter paper and placed in a climate controlled room (17°C, 60% relative humidity, 12:12 light:dark). Every fourth day, the weights of the larvae were measured and moved to a new Petri dish containing a fresh leaf. Finally, at pupation, we measured the weight of the individual pupa.

### 2.7 Olfactory preferences of *Asecodes lucens*

We examined the ability of 2‐day‐old gravid *A. lucens* females from both natal origins to discriminate between olfactory cues emitted by either damaged plants or plants with larvae in glass Y‐tube olfactometers (Humi Glas AB, Sweden). The stem of the Y‐tube (ø 18 mm × 10 cm) ended in two arms separated in a 90° angle (ø 18 mm × 7.5 cm) in which odors were delivered through an airflow at 20 ml/s. The odor treatments were placed in gas‐washing bottles that were connected to the Y‐tube with Teflon tubing (ø 5 mm) following the same protocol as described for the two‐armed olfactometers (section 2.3). The experiments were run under low light conditions produced by diffuse LED lights placed 50 cm above the Y‐tubes, and the position of the gas‐washing bottles and the Y‐tubes was specifically arranged to eliminate any visual cues that could guide the movement of the parasitoids. At the start of the experiment, an individual parasitoid was placed into the Y‐tube and given 1 min to acclimatize. We recorded a choice when the parasitoid passed two‐thirds of a Y‐tube arm and stayed there for at least 5 s. All parasitoids that did not make a decisive choice after 5 min were disregarded in the analysis (following Stenberg, Heijari, Holopainen, and Ericson (2007)).

We firstly tested the olfactory response of parasitoids of both natal origins to olfactory cues emitted by either damaged host plants or by larvae feeding on host plants, vs. a control of humidified air. These tests indicated that the parasitoids were only attracted to feeding‐damaged plants with larvae on them; thus, we excluded feeding‐damaged plants without larvae from further experiments. In the second run of experiments, we tested whether parasitoids of both natal origins were able to distinguish between olfactory cues of larvae on either host plant in two‐test odor experiments (Figure 7). The larval treatments were represented by six early instar *G. sagittariae* larvae that were allowed to feed on the host plants for 12–24 hr prior to the experiment. Shortly before the experiment, shoots of 10–15 cm containing the larvae were detached from the plant and used in the experiments. Each Y‐tube was used for three consecutive tests after which it was cleaned with an odorless detergent and ethanol and dried in an oven at 200°C to eliminate odor cues.

### 2.8 Performance of *Asecodes lucens*

To determine the performance of *A. lucens* on larvae developing on both host plant, we used field‐collected *G. sagittariae* larvae from field locations dominated by either host plant species. Upon collection, it is not possible to determine which larvae are parasitized and, therefore, the larvae were fed until pupation after which the parasitized (e.g., mummified) pupae were maintained in 1.5‐ml plastic tubes under controlled conditions (12°C, 60% RH, 12:12 L:D). All mummies were monitored, and adult parasitoids that emerged were removed and stored in ethanol for further analysis. We counted the number of parasitoids emerging from each mummy, and for each individual, we determined the sex and measured the length of the hind tibia under a stereo microscope as explained in Stenberg and Hambäck (2010).

### 2.9 Statistical analysis

The olfactory preference of *G. sagittariae* was analyzed using logistic regressions with a quasi‐binomial distribution to account for overdispersion. For the one‐test odor experiments, we compared the preference for the different odor treatments using feeding damage, natal origin and the host plant stimulus as explanatory variables. For the two‐test odor experiments, we only used the natal origin of the beetle as an explanatory variable as the plant material was selected not to differ in feeding damage. Subsequently, to determine whether the olfactory preferences could be explained by differences in the volatile emission of both plant species, we compared the area under the GC‐MS peaks of all EAD‐active compounds using the adonis function of the vegan package (Oksanen et al., 2016) with the plant species as an explanatory variable. The adonis function is a multivariate version of an ANOVA that uses permutations to partition the data matrix (i.e., GC‐MS peak area per compounds) between the two host plant species. For one compound (two replicates γ‐caprolactone), emissions were recorded as trace compounds in the GC‐MS recording, thus being too small for quantification. In these cases, the area under the curve cannot be quantified and we assigned a small value (100.000), which roughly corresponds to a magnitude of ten times smaller than quantifiable peaks of γ‐caprolactone. We corrected the area under the peak with the feeding damage of *G. sagittariae* on all leaves from that specific recording before log‐transforming the values. We added a value corresponding to half the area under the smallest peak to each value in the data set before transformation to prevent problems with zero values (i.e., compounds without emission values). We performed an indicator species analysis using the indval function of the labdsv package as a post hoc test (Roberts, 2016). Finally, nonmetric multidimensional scaling (NMDS) was used to quantify and visualize compositional similarity of the emission of compounds by both host plant species.

To determine the oviposition preference of *G. sagittariae* females, we compared the relative number of egg batches and the mean number of eggs per batch using generalized linear mixed model (GLMM) with a Poisson distribution including the oviposition plant choice, the natal origin of beetles, the host plant frequency (i.e., either 0, 0.25, 0.50, 0.75, or 1), and the necessary interactions as explanatory variables. We first analyzed the complete data set to determine which factor affected the oviposition behavior of *G. sagittariae* females, and subsequently, we ran the same analysis on subsets for both natal origins. In both analyses, we included the individual cage as a random variable to estimates relative rather than the absolute number of batches or eggs, by correcting for the number of host plant species in the patch. For the larval performance experiments, we estimated the growth rate of each individual larva by fitting a linear regression through the log‐transformed weights measured at the different developmental time points. We then used the estimated growth rates in a linear model with natal origin, oviposition plant, and an interaction between the two variables as explanatory variables using a GLM. Subsequently, we used a similar model to analyze the pupae weights.

The olfactory preference of the parasitoids in the one‐test odor experiments was determined using a GLM, with a binomial error distribution, including the natal origin, the odor source, and the necessary interaction as explanatory variables. For the two‐test odor experiments, only the natal origin was included as an explanatory variable. Furthermore, to explore differences in the performance of *A. lucens*, we first performed a GLM on brood size with natal origin and mummy length as explanatory variables. Secondly, we analyzed the hind tibia length with a GLM with the natal origin, mummy length, sex and brood size as explanatory variables. Finally, the sex ratio was analyzed with a GLM with binomial distribution accounting for the natal origin, mummy length, and brood size.

Prior to all analyses, Fligner–Killeen tests were used to check for homogeneity of variances. These tests showed that for the beetle larval performance data a log transformation was necessary before analyzing the data further. After each analysis, the normality of the residuals and the Q‐Q plots were checked through visual inspection. All analyses were carried out in R (v. 3.2.3; R Foundation for Statistical Computing, Vienna, AT). For those analyses necessary, the lme4 package (Bates et al. 2013) was used for fitting the mixed effect models and the car package (Fox & Weisber, 2011) was used for the likelihood ratio tests.

## 3 Results

### 3.1 Olfactory preferences of *Galerucella sagittariae*

In the one‐test odor experiments, *G. sagittariae* showed a preference toward odors emitted by damaged host plants (*χ* ^2^ = 13.2, *p* < .001; Figure 2), but not by undamaged plants (Figure S1), and this preference was not affected by the identity of the host plant or the natal origin of the beetle. In the two‐test odor experiments, natal *Lysimachia* beetles showed a preference for *L. thyrsiflora*, whereas natal *Potentilla* beetles showed no preference to either host plant (Figure 2; Table S1).

### 3.2 Responses to headspace of host plants

**Figure 4:**
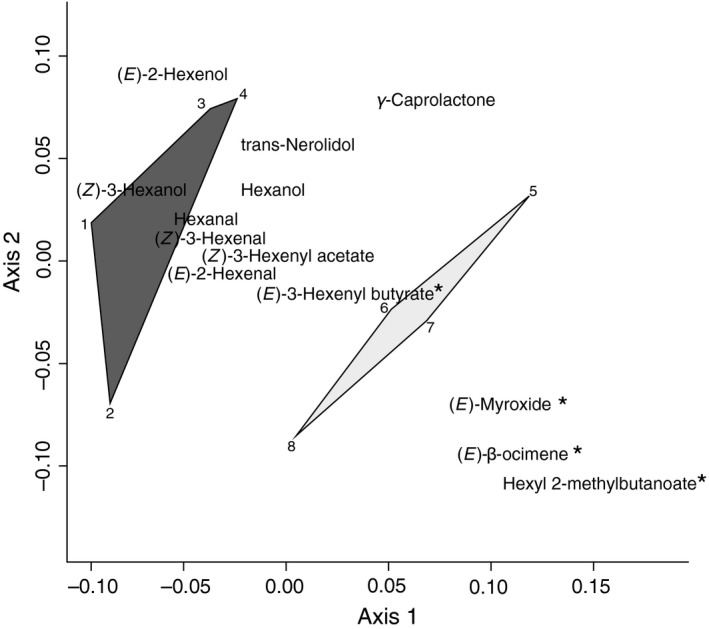
Nonmetric multidimensional scaling (NMDS) plot for the GC‐EAD‐active compounds present in *Potentilla palustris* (light gray: *n* = 4) and *Lysimachia thyrsiflora* (dark gray: *n* = 4) headspaces. The analysis was based on the log‐transformed area underneath the GC‐MS peak of each compound (*p < .05)

The GC‐EAD showed that multiple olfactory compounds from the headspace of the damaged host plants caused responses in *G. sagittariae* antennae. Among the EAD‐active compounds, both host plants emitted quantifiable amounts in their headspace, with the exception of hexyl 2‐methylbutanoate, which was not found in *L. thyrsiflora* (Figure 3; Table S2). The multivariate analysis showed that the amounts of the GC‐EAD‐active compounds differed between the host plants (adonis; F_1,6_ = 5.9, *p* = .019), and the species indicator analysis found significant differences between host plants in the emission of (*E*)‐β‐ocimene (*p* = .025), hexyl 2‐methylbutanoate (*p* = .029), (*E*)‐3‐hexenylbutyrate (*p* = .036), and (*E*)‐myroxide (*p* = .042). These compounds were all produced in higher amounts by *P. palustris* than by *L. thyrsiflora* (Figure 4).

### 3.3 Oviposition preference of *Galerucella sagittariae*

**Figure 5:**
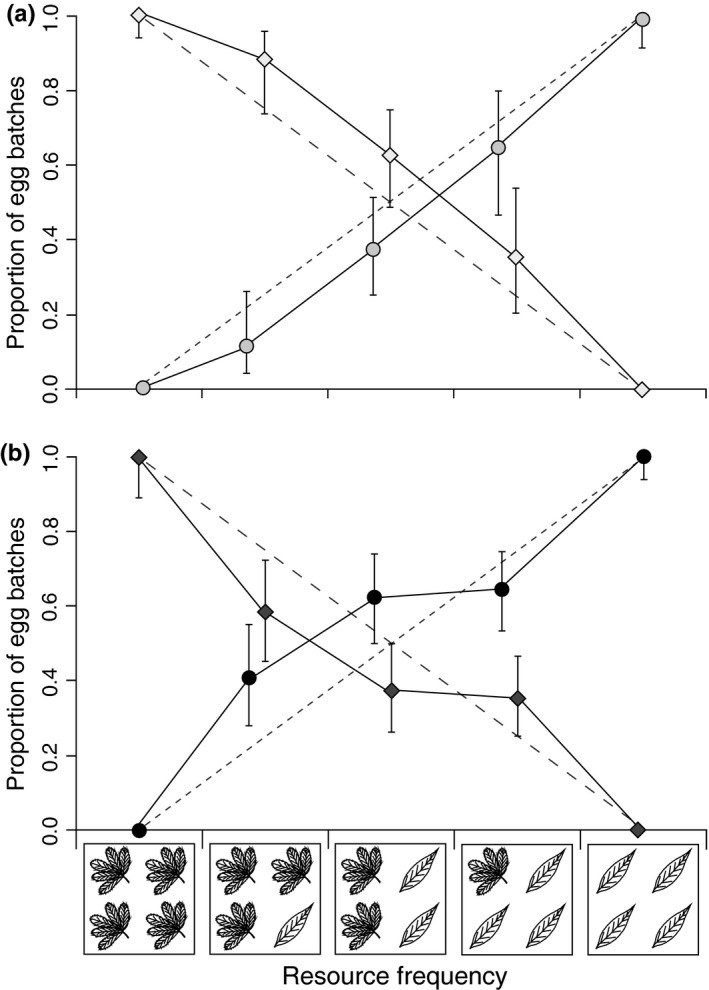
The proportion of egg batches (± 95% C.I.) laid by natal *Potentilla* beetles (a: *n* = 5) and natal *Lysimachia* beetles (b: *n* = 10) on *P*. *palustris* (squares) and *L*. *thyrsiflora* (circles). The dashed lines give the expected proportion of egg batches on *P*. *palustris*‐ and *L*. *thyrsiflora‐*based plant frequency (cf. boxes below the *x*‐axis)

**Figure 6:**
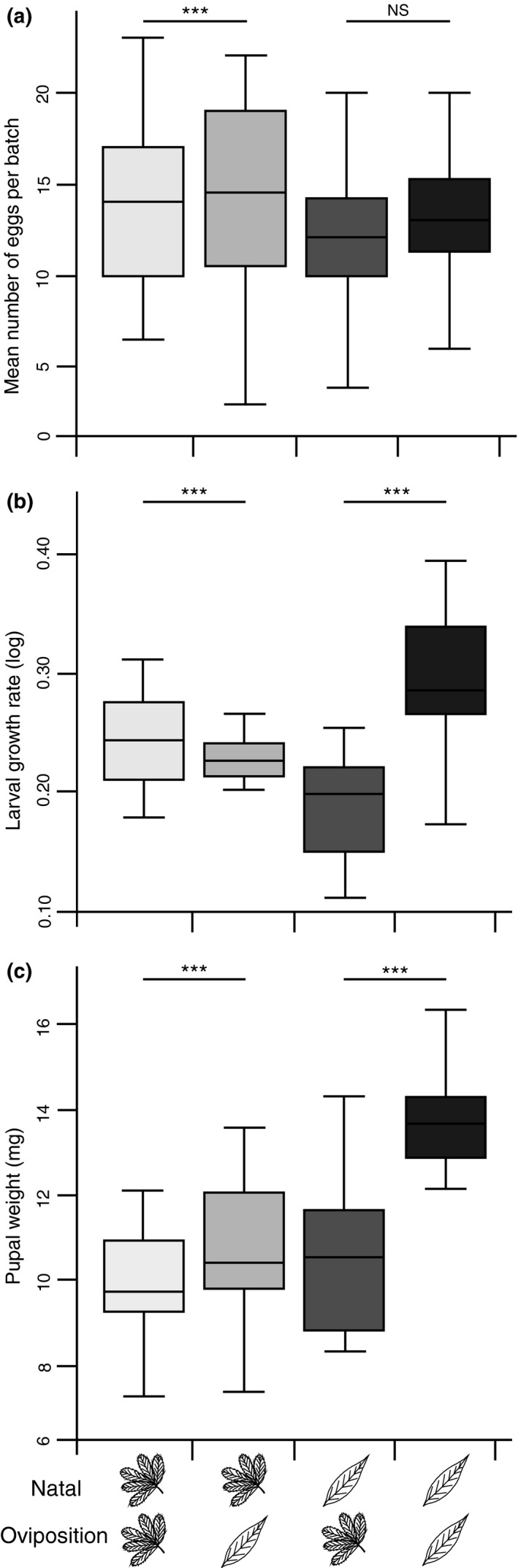
Number of eggs per egg batches (a), larval growth rate (b), and pupal weight (c) of natal *Potentilla* beetles and natal *Lysimachia* beetles on *P. palustris* and *L*. *thyrsiflora* (*n* = 20 for each treatment). The difference between oviposition plants for both natal origins was tested with planned comparisons (***p < .001)

The number of egg batches was affected by oviposition plant species (i.e., female oviposition choice), the natal origin of the female and interactive effects of resource frequency and natal origin, resource frequency and oviposition plant, and a three‐way interaction between those three variables (Table 1). For natal *Potentilla* beetles, the oviposition on the host plants was roughly proportional to the host plant frequency suggesting no selection between host plants (Figure 5a). The natal *Lysimachia* beetles, on the other hand, showed a preference toward *L. thyrsiflora* irrespective of the host plant frequency (Figure 5b). Furthermore, the mean number of eggs per egg batch was affected by natal origin and oviposition plant, but not by their interaction. Natal *Potentilla* beetles laid a higher number of eggs per batch than natal *Lysimachia* beetles, and the beetles of both natal origins laid egg batches with a higher mean number of eggs on *L. thyrsiflora* (Figure 6a; Table 1).

**Table 1:** Likelihood ratio tests (*X* ^*2*^ *)* for the oviposition preference of *Galerucella sagittariae*. The natal origin corresponds to the host plant species from which the *G*. *sagittariae* females derived, and the oviposition plant corresponds to the host plant species on which the eggs were laid. The frequency was based on the proportion of *Potentilla palustris* plants in the cage

Natal origin	Variables	Batches			Eggs		
		*X* ^*2*^	*df*	*p*	*X* ^*2*^	*df*	*p*
*Potentilla* and *Lysimachia*	Natal origin	12.47	1	<.001	27.19	1	<.001
	Oviposition plant	0.20	1	.652	7.96	1	.004
	Frequency	0.15	1	.699	0.24	1	.624
	Frequency *×* Natal origin	4.29	1	.038	0.18	1	.667
	Frequency *×* Oviposition plant	10.26	1	.001	2.75	1	.097
	Frequency *×* Natal origin *×* Oviposition plant	9.86	1	.001	0.51	1	.475
*Potentilla*	Oviposition plant	1.74	1	.187	7.16	1	.007
	Frequency	0.06	1	.812	1.62	1	.203
	Frequency *×* Oviposition plant	0.53	1	.465	0.17	1	.679
*Lysimachia*	Host plant	0.93	1	.336	1.59	1	.207
	Frequency	1.52	1	.217	1.99	1	.158
	Frequency *×* Oviposition plant	7.99	1	.005	0.49	1	.484

### 3.4 Performance of *Galerucella sagittariae* larvae

The larval growth rate of *G. sagittariae* was affected by the oviposition plant (i.e., current host plant of larvae), and by an interaction between the natal origin and the oviposition plant (Table 2). While the larval growth rates for both natal *Lysimachia* and natal *Potentilla* beetles were higher on its natal host plant, the difference in growth rates between the oviposition plants was much higher for natal *Lysimachia* beetles compared with natal *Potentilla* beetles (Figure 6b). The pupae weight was affected by the natal origin, the oviposition plant and by an interaction between the two. Irrespective of the natal origin, the pupae weights on *L. thyrsiflora* were higher than on *P. palustris*, and the interaction occurred because the weights of natal *Lysimachia* beetles differed more between host plants than the weights of natal *Potentilla* beetles did (Table 2; Figure 6c).

**Table 2:** Likelihood ratio tests (*X* ^*2*^ *)* for the larval performance and pupae weight of *Galerucella sagittariae*. The natal origin corresponds to the host plant species from which the *G*. *sagittariae* females derived, and the oviposition plant corresponds to the host plant species on which the eggs were laid

Stage	Variables	*X* ^*2*^	*df*	*p*‐Value
Larvae	Natal origin	2.04	1	.153
	Oviposition plant	41.78	1	<.001
	Natal origin *×* Oviposition plant	67.51	1	<.001
Pupae	Natal origin	25.71	1	<.001
	Oviposition plant	34.72	1	<.001
	Natal origin *×* Oviposition plant	11.93	1	<.001

### 3.5 Olfactory preferences of *Asecodes lucens*

**Figure 7:**
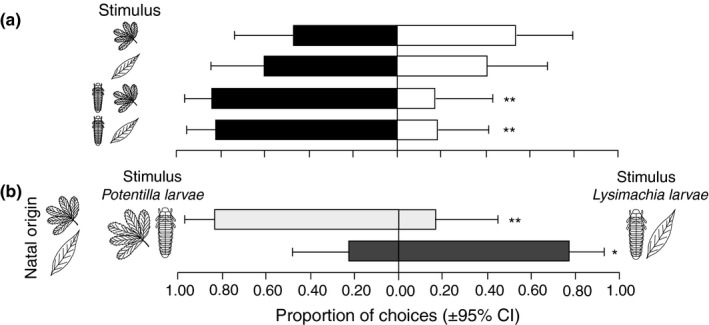
(a) Behavioral response of *Asecodes lucens* (± 95% C.I.) to odors of damaged host plants (*n* = 15 for both treatments), to larvae on *Potentilla palustris* (*n* = 22) and *Lysimachia thyrsiflora* (*n* = 18) vs. control treatments (black bars) consisting of humidified air. (b) Behavioral responses of natal *Potentilla* parasitoids (*n* = 18) and natal *Lysimachia* parasitoids (*n* = 18) in two‐test odor treatments. (*p < .05, **p < .01)

*Asecodes lucens* preferred *G. sagittariae* larvae in combination with either *P. palustris* or *L. thyrsiflora* as the host plant over the control treatment of humidified air (GLM; χ^2^ = 7.1, *p* < .01), but showed no preference to volatiles from damaged plants without larvae over the control treatment (Figure 7; Table S3). The strength of the attraction was not affected by the identity of the host plant*,* or by the combination of *G. sagittariae* larvae and host plant (Figure 7). On the other hand, in the two‐test odor experiment *A. lucens* showed a preference for the combination of larvae and host plant of the natal origin (GLM; χ^2^ = 14.5, *p* < .001; Figure 7; Table S3).

### 3.6 Performance of *Asecodes lucens*

**Figure 8:**
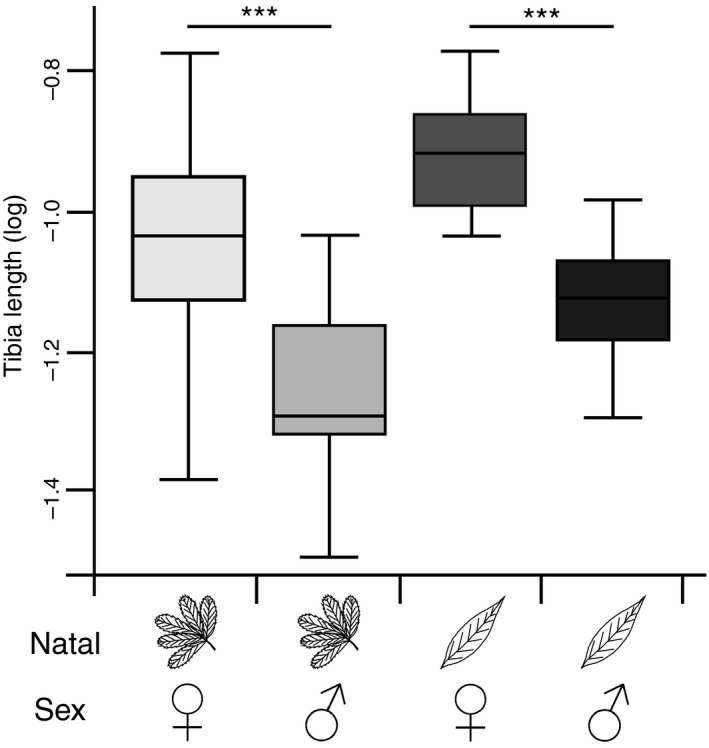
The log‐transformed hind tibia length for female and male *Asecodes lucens* hatching from *Galerucella larvae* from *Potentilla palustris* (light gray) and *Lysimachia thyrsiflora* (dark grays). The difference between oviposition plants for both natal origins was tested with planned comparisons (**p < .01, **p < .001)

The length of mummified larvae (GLM; $\chi_{1,39}^{2}$ = 0.14, *p* = .714), and brood size of parasitoids emerging from the mummies were not different between larvae of different natal origin (Table 3). However, the sex ratio from broods originating from *Lysimachia* larvae (23% males, 76% females) was more strongly biased toward females than broods from *Potentilla* larvae (39% males, 61% females), leading to a significant effect of natal origin on sex ratio. Furthermore, the hind tibia length was longer in females (Figure 8) and, from broods originating from *Lysimachia* larvae (Table 3), and was positively correlated with the length of the mummy (Figure S2).

**Table 3:** Likelihood ratio tests (*X* ^*2*^ *)* of the performance related traits of *Asecodes lucens*. The natal origin corresponds to the combination of *G*. *sagittariae* larvae and natal host plant species of *A*. *lucens*

Response variable	Variables	*X* ^*2*^	*df*	*p*
Brood size	Natal origin	0.36	1	.546
	Mummy length	0.30	1	.582
	Natal origin *×* Mummy length	0.34	1	.558
Sex ratio	Brood size	0.33	1	.563
	Natal origin	4.26	1	.039
	Mummy length	1.09	1	.296
	Brood size *×* Natal origin	0.16	1	.691
	Natal origin *×* Mummy length	1.28	1	.258
Hind tibia length	Brood size	2.35	1	.126
	Sex	98.67	1	<.001
	Natal origin	26.18	1	<.001
	Mummy length	4.04	1	.044
	Brood size *×* Natal origin	1.83	1	.176
	Natal origin *×* Mummy length	0.56	1	.453
	Natal origin *×* Mummy length *×* Sex	0.52	2	.771

## 4 Discussion

Our results showed that the relationship between oviposition preference and larval performance depends on the interplay between natal origin and the quality of the current host for both the herbivorous *Galerucella sagittariae* and its larval parasitoid *Asecodes lucens*. However, the interaction between natal origin and current host caused different patterns in the oviposition preference and larval performance relationships for the two insect species. For the herbivore, only those females that originated from *Lysimachia thyrsiflora* maintained an olfactory and oviposition preference for their natal host plant (Figures 2 and 5), while also having higher larval performance on the natal host plant. The females originating from *Potentilla palustris,* on the other hand, showed no preference for their natal host plant and the larval performance was also lower on *P. palustris* (Figure 6). For the parasitoids, the natal origin strongly affected the olfactory preference toward the combination of odors from the larvae they originated from and their respective host plant (Figure 7). However, the larger body size of *A. lucens* offspring and the skewed sex ratio toward females originating from natal *Lysimachia* larvae suggest a reproductive advantage for parasitoids developing on natal *Lysimachia* larvae (Figures 8 and S2). Overall, the only positive relationship between oviposition preference and larval performance occurred for natal *Lysimachia* beetles, while neither natal *Potentilla* beetles nor any of the parasitoids showed such a relationship.

Several studies suggest that a lack of preference–performance relationship cannot simply be explained by a mismatch between female preference and host plant quality (Gripenberg et al., 2010; Jaenike, 1978; Sutter & Kawecki, 2009). A lack of this relationship may also be due to other factors influencing the female fitness (Cornell & Hawkins, 2003; Jaenike, 1978; Stamps et al., 2009; Thompson, 1988) or due to neural constraints (Bernays, 2001; Bruce & Pickett, 2011; Cunningham, 2012). In our system, it was evident that the natal host was often a better predictor of female preference than of larval performance. This outcome suggests that in our tritrophic system the constraint in locating any host is more limiting for female fitness than the specific host quality. It is likely that the females experience a chemical legacy effect from their natal host that affect host finding and underlie the observed behavioral responses, as has been shown for other systems (Corbet, 1985; van Emden et al., 1996; Godfray, 1994). We identified potential compounds underlying host preference and found that hexyl 2‐methylbutanoate was absent in *L. thyrsiflora,* whereas (*E*)‐β‐ocimene, (*E*)‐myroxide, and (*E*)‐3‐hexenylbutyrate were all released in higher quantities by *P. palustris* (Figures 3a and 4). While it is likely that the presence of hexyl 2‐methylbutanoate and the higher quantities of the three other compounds in *P. palustris* caused natal *Lysimachia* beetles not to recognize *P. palustris* as an alternative host, it is interesting to observe that the absence of hexyl 2‐methylbutanoate in *L. thyrsiflora* did not result in natal *Potentilla* beetles to reject *L. thyrsiflora* as a host plant. Moreover, recent work on *Asecodes parviclava* (Thomson), a species closely related to *A. lucens,* suggested that (*E*)‐β‐ocimene and hexyl 2‐methylbutanoate may also be involved in the host selection by the parasitoids (Fors, L., Mozūraitis, R., Blažytė‐Čereškienė, L., Verschut, T.A. & Hambäck, P.A., unpublished), which corresponds to the hypothesis that herbivores and their natural enemies use similar chemical information to locate suitable hosts (Fatouros et al., 2012; Takabayashi et al., 1998; Vet & Dicke, 1992).

Other leaf beetle species have been found to select suboptimal host plants with high levels of defense chemicals (Denno et al., 1990; Häggström & Larsson, 1995), and the suggested mechanism behind this selection is that these plants provide lower parasitism pressure (Denno et al., 1990; Stamp, 2001). Although our current study was not specifically designed to test this hypothesis, our data may provide a starting point for testing the possibility that herbivore host selection is also determined by enemy‐free space. It is notable for this hypothesis that the chemical legacy seemed to be stronger in the host selection by the parasitoids compared with the herbivore hosts. More specifically, the parasitoids hatching from both larval species showed a strong preference for the olfactory cues derived from the combination of their natal larval‐ and respective host plant, without responding to odors from the host plants without larvae (Figure 7). We may hypothesize that such a strong chemical legacy in the host search by the parasitoid would have the consequence that it is advantageous for their herbivore host to actually switch host plants and not show a strong legacy effect. If this is true, it may thus be advantageous for the herbivore species to maintain multiple host plants in their diet, thus selecting against specialization.

This suggestion is of special interests for the interactions in our tritrophic system as they can help to understand the evolutionary mechanism behind possible host speciation processes for the beetles. Assuming that the natal *Lysimachia* parasitoids maintained strongly biased host selection over multiple generations, this high parasitism pressure may have caused *G*. *sagittariae* to switch to the lower‐quality *P*. *palustris* as an alternative host plant (Ballabeni et al., 2001; Denno et al., 1990; Stamp, 2001). This alternative explanation could then indicate why the difference in larval performance was not large enough to prevent natal *Potentilla* beetles from using the lower‐quality *P*. *palustris* (Figures 5 and 6), and why natal *Potentilla* beetles responded to odors from *L*. *thyrsiflora* (Figure 2). For other *Galerucella* beetles, host race formation has frequently been documented (Ikonen, Sipura, Miettinen, & Tahvanainen, 2003; Nokkala & Nokkala, 1998; Stenberg & Axelsson, 2008), and such processes would explain our current observations. For further research, it would be of interest to collect long‐term data on host use across generations and whether observed host uses also has resulted in population differentiation. Such data would show if the results found in our study are mainly based on natal experience, or if there are also due to evolutionary changes. Overall, our study suggests that our general knowledge of host use in tritrophic systems can be improved by incorporating information on both host preference and larval performance for all interacting species. Understanding the preference–performance relationships for both the plant host–herbivore and insect host–parasitoid interactions can, consequently, serve as a possible explanation why herbivorous beetles adapt to lower‐quality resources, and help understand how such interaction could possibly lead to host race formation (Pappers et al., 2002; Pfennig et al., 2010; Via, 1999), or sympatric speciation (Beltman et al., 2004; Bernays, 2001).

## Conflict of Interest

None declared.

## Author contribution

The study and experiments were designed by TAV and PAH and performed by TAV. RM performed odor collections and chemical identification; LBC and VA performed GC‐EAD recordings and additional chemical identification. The statistical analyses were performed by TAV and PAH, and the manuscript was written by TAV and PAH with comments from the other authors.

## References

[ece32826-bib-0001] Ballabeni, P. , Wlodarczyk, M. , & Rahier, M. (2001). Does enemy‐free space for eggs contribute to a leaf beetle's oviposition preference for a nutritionally inferior host plant? Functional Ecology, 15, 318–324.

[ece32826-bib-0501] Bates, D. , Maechler, M. , Bolker, B. , & Walker, S. (2013). Fitting linear mixed‐effects models using lme4. Journal of Statistical Software, 67, 1–48.

[ece32826-bib-0002] Beltman, J. B. , Haccou, P. , & ten Cate, C. (2004). Learning and colonization of new niches: A first step toward speciation. Evolution, 58, 35–46. 15058717 10.1111/j.0014-3820.2004.tb01571.x

[ece32826-bib-0003] Bernal, J. S. , Luck, R. F. , & Morse, J. G. (1998). Sex ratios in field populations of two parasitoids (Hymenoptera: Chalcidoidea) of *Coccus hesperidum* L. (Homoptera: Coccidae). Oecologia, 116, 510–518. 28307520 10.1007/s004420050616

[ece32826-bib-0004] Bernays, E. A. (1988). Host specificity in phytophagous insects: Selection pressure from generalist predators. Entomologia Experimentalis et Applicata, 49, 131–140.

[ece32826-bib-0005] Bernays, E. A. (2001). Neural limitations in phytophagous insects: Implications for diet breadth and evolution of host affiliation. Annual Review of Entomology, 46, 703–727. 10.1146/annurev.ento.46.1.703 11112184

[ece32826-bib-0006] Bruce, T. J. A. , & Pickett, J. A. (2011). Perception of plant volatile blends by herbivorous insects ‐ finding the right mix. Phytochemistry, 72, 1605–1611. 21596403 10.1016/j.phytochem.2011.04.011

[ece32826-bib-0007] Charnov, E. , Los‐den Hartogh, R. L. , Jones, W. T. , & van den Assem, J. (1981). Sex ratio evolution in a variable environment. Nature, 289, 27–33. 7453809 10.1038/289027a0

[ece32826-bib-0008] Corbet, S. A. (1985). Insect chemosensory responses: A chemical legacy hypothesis. Ecological Entomology, 10, 143–153.

[ece32826-bib-0009] Cornell, H. V. , & Hawkins, B. A. (2003). Herbivore responses to plant secondary compounds: A test of phytochemical coevolution theory. The American Naturalist, 161, 507–522. 10.1086/368346 12776881

[ece32826-bib-0010] Cunningham, J. P. (2012). Can mechanism help explain insect host choice? Journal of Evolutionary Biology, 25, 244–251. 22225990 10.1111/j.1420-9101.2011.02435.x

[ece32826-bib-0011] Davis, J. M. (2008). Patterns of variation in the influence of natal experience on habitat choice. The Quarterly Review of Biology, 83, 363–380. 19143336 10.1086/592851

[ece32826-bib-0012] Davis, J. M. , & Stamps, J. A. (2004). The effect of natal experience on habitat preferences. Trends in Ecology & Evolution, 19, 411–416. 16701298 10.1016/j.tree.2004.04.006

[ece32826-bib-0013] Denno, R. F. , Larsson, S. , & Olmstead, K. L. (1990). Role of enemy‐free space and plant quality in host‐plant selection by willow beetles. Ecology, 71, 124–137.

[ece32826-bib-0014] Dolgin, M. M. (1979). *Asecodes mento* Walker (Hymenoptera, Chalcidoidea, Eulophidae), a parasite of the water‐lily beetle (*Galerucella nymphaeae*) in the Altai. Entomological Review, 58, 145–147.

[ece32826-bib-0015] Dukas, R. , & Duan, J. J. (2000). Potential fitness consequences of associative learning in a parasitoid wasp. Behavioral Ecology, 11, 536–543.

[ece32826-bib-0016] Ehrlich, P. R. , & Raven, P. H. (1964). Butterflies and plants: A study in coevolution. Evolution, 18, 586–608.

[ece32826-bib-0017] van Emden, H. F. , Sponagl, B. , Wagner, E. , Baker, T. , Ganguly, S. , & Douloumpaka, S. (1996). Hopkins’ ‘host selection principle’, another nail in its coffin. Physiological Entomology, 21, 325–328.

[ece32826-bib-0018] Fatouros, N. E. , Lucas‐Barbosa, D. , Weldegergis, B. T. , Pashalidou, F. G. , van Loon, J. J. A. , Dicke, M. , … Huigens, M. E. (2012). Plant volatiles induced by herbivore egg deposition affect insects of different trophic levels. PLoS ONE, 7, e43607. 22912893 10.1371/journal.pone.0043607 PMC3422343

[ece32826-bib-0019] Fournet, S. , Poinsot, D. , Brunel, E. , Nenon, J. P. , & Cortesero, A. M. (2001). Do female coleopteran parasitoids enhance their reproductive success by selecting high‐quality oviposition sites? Journal of Animal Ecology, 70, 1046–1052.

[ece32826-bib-0020] Fox, J. , & Weisber, S. (2011). An (R) companion to applied regression, 2nd ed Thousand Oaks, CA: Sage Publications.

[ece32826-bib-0021] Fritz, R. S. , Crabb, B. A. , & Hochwender, C. G. (2000). Preference and performance of a gall‐inducing sawfly: A test of the plant vigor hypothesis. Oikos, 89, 555–563.

[ece32826-bib-0022] Godfray, H. C. J. (1994). Parasitoids: Behavioral and evolutionary ecology. Princeton, NJ: Princeton University Press.

[ece32826-bib-0023] Gripenberg, S. , Mayhew, P. J. , Parnell, M. , & Roslin, T. (2010). A meta‐analysis of preference–performance relationships in phytophagous insects. Ecology Letters, 13, 383–393. 20100245 10.1111/j.1461-0248.2009.01433.x

[ece32826-bib-0024] Häggström, H. , & Larsson, S. (1995). Slow larval growth on a suboptimal willow results in high predation mortality in the leaf beetle *Galerucella lineola* . Oecologia, 104, 308–315. 28307587 10.1007/BF00328366

[ece32826-bib-0025] Hambäck, P. A. , Pettersson, J. , & Ericson, L. (2003). Are associational refuges species‐specific? Functional Ecology, 17, 87–93.

[ece32826-bib-0026] Harvey, J. A. , Gols, R. , Snaas, H. , Malcicka, M. , & Visser, B. (2015). Host preference and offspring performance are linked in three congeneric hyperparasitoid species. Ecological Entomology, 40, 114–122.

[ece32826-bib-0027] Harvey, J. A. , van Dam, N. M. , & Gols, R. (2003). Interactions over four trophic levels: Foodplant quality affects development of a hyperparasitoid as mediated through a herbivore and its primary parasitoid. Journal of Animal Ecology, 72, 520–531.

[ece32826-bib-0028] Hilker, M. , & Meiners, T. (2011). Plants and insect eggs: How do they affect each other? Phytochemistry, 72, 1612–1623. 21439598 10.1016/j.phytochem.2011.02.018

[ece32826-bib-0029] Hippa, H. , & Koponen, S. (1976). Distribution of the species of *Galerucella* (Col., Chrysomelidae) on cloudberry in Fennoscandia. Reports from the Kevo Subarctic Research Station, 13, 40–43.

[ece32826-bib-0030] Ikonen, A. , Sipura, M. , Miettinen, S. , & Tahvanainen, J. (2003). Evidence for host race formation in the leaf beetle *Galerucella lineola* . Entomologia Experimentalis et Applicata, 108, 179–185.

[ece32826-bib-0031] Immelmann, K. (1975). Ecological significance of imprinting and early learning. Annual Review of Ecology and Systematics, 6, 15–37.

[ece32826-bib-0032] Jaenike, J. (1978). On optimal oviposition behavior in phytophagous insects. Theoretical Population Biology, 14, 350–356. 751265 10.1016/0040-5809(78)90012-6

[ece32826-bib-0033] Mayhew, P. J. (1997). Adaptive patterns of host‐plant selection by phytophagous insects. Oikos, 79, 417–428.

[ece32826-bib-0034] Mitchell, R. (1981). Insect behavior, resource exploitation, and fitness. Annual Review of Entomology, 26, 373–396.

[ece32826-bib-0035] Mooney, K. A. , Pratt, R. T. , & Singer, M. S. (2012). The tri‐trophic interactions hypothesis: Interactive effects of host plant quality, diet breadth and natural enemies on herbivores. PLoS ONE, 7, e34403. 22509298 10.1371/journal.pone.0034403 PMC3324533

[ece32826-bib-0036] Nokkala, C. , & Nokkala, S. (1998). Species and habitat races in the chrysomelid *Galerucella nymphaeae* species complex in northern Europe. Entomologia Experimentalis et Applicata, 89, 1–13.

[ece32826-bib-0037] van Nouhuys, S. , Reudler, J. H. , Biere, A. , & Harvey, J. A. (2012). Performance of secondary parasitoids on chemically defended and undefended hosts. Basic and Applied Ecology, 13, 241–249.

[ece32826-bib-0038] Oksanen, J. , Blanchet, F. G. , Kindt, R. , Legendre, P. , Minchin, P. R. , O'Hara, R. B. , … Wagner, H. (2016). vegan: Community Ecology Package. R package version 2.3‐5.

[ece32826-bib-0039] Pappers, S. M. , van der Velde, G. , & Ouborg, J. (2002). Host preference and larval performance suggest host race formation in *Galerucella nymphaeae* . Oecologia, 130, 433–440. 10.1007/s00442-001-0822-3 28547051

[ece32826-bib-0040] Pfennig, D. W. , Wund, M. A. , Snell‐Rood, E. C. , Cruickshank, T. , Schlichting, C. D. , & Moczek, A. P. (2010). Phenotypic plasticity's impacts on diversification and speciation. Trends in Ecology & Evolution, 25, 459–467. 20557976 10.1016/j.tree.2010.05.006

[ece32826-bib-0041] Price, P. W. , Bouton, C. E. , Gross, P. , McPheron, B. A. , Thompson, J. N. , & Weis, A. E. (1980). Interactions among three trophic levels: Influence of plants on interactions between insect herbivores and natural enemies. Annual Review of Ecology and Systematics, 11, 41–65.

[ece32826-bib-0042] Randlkofer, B. , Obermaier, E. , Hilker, M. , & Meiners, T. (2010). Vegetation complexity—the influence of plant species diversity and plant structures on plant chemical complexity and arthropods. Basic and Applied Ecology, 11, 383–395.

[ece32826-bib-0043] Roberts, D. W. (2016). labdsv: Ordination and Multivariate Analysis for Ecology. R package version 1.8.

[ece32826-bib-0044] Singer, M. C. (1983). Determinants of multiple host use by a phytophagous insect population. Evolution, 37, 389–403. 10.1111/j.1558-5646.1983.tb05547.x 28568358

[ece32826-bib-0045] Stamp, N. (2001). Enemy‐free space via host plant chemistry and dispersion: Assessing the influence of tri‐trophic interactions. Oecologia, 128, 153–163. 10.1007/s004420100679 28547463

[ece32826-bib-0046] Stamps, J. , Luttbeg, B. , & Krishnan, V. V. (2009). Effects of survival on the attractiveness of cues to natal dispersers. The American Naturalist, 173, 41–46. 10.1086/593306 19090706

[ece32826-bib-0047] Stenberg, J. A. , & Axelsson, E. P. (2008). Host race formation in the meadowsweet and strawberry feeding leaf beetle *Galerucella tenella* . Basic and Applied Ecology, 9, 560–567.

[ece32826-bib-0048] Stenberg, J. A. , & Hambäck, P. A. (2010). Host species critical for offspring fitness and sex ratio for an oligophagous parasitoid: Implications for host coexistence. Bulletin of Entomological Research, 100, 735–740. 20626928 10.1017/S0007485310000143

[ece32826-bib-0049] Stenberg, J. A. , Heijari, J. , Holopainen, J. K. , & Ericson, L. (2007). Presence of *Lythrum salicaria* enhances the bodyguard effects of the parasitoid *Asecodes mento* for *Filipendula ulmaria* . Oikos, 116, 482–490.

[ece32826-bib-0050] Sutter, M. , & Kawecki, T. J. (2009). Influence of learning on range expansion and adaptation to novel habitats. Journal of Evolutionary Biology, 22, 2201–2214. 19824931 10.1111/j.1420-9101.2009.01836.x PMC2779469

[ece32826-bib-0051] Takabayashi, J. , Sato, Y. , Horikoshi, M. , Yamaoka, R. , Yano, S. , Ohsaki, N. , & Dicke, M. (1998). Plant effects on parasitoid foraging: Differences between two tritrophic systems. Biological Control, 11, 97–103.

[ece32826-bib-0052] Tholl, D. , Boland, W. , Hansel, A. , Loreto, F. , Röse, U. S. R. , & Schnitzler, J. P. (2006). Practical approaches to plant volatile analysis. The Plant Journal, 45, 540–560. 16441348 10.1111/j.1365-313X.2005.02612.x

[ece32826-bib-0053] Thompson, J. N. (1988). Evolutionary ecology of the relationship between oviposition preference and performance of offspring in phytophagous insects. Entomologia Experimentalis et Applicata, 47, 3–14.

[ece32826-bib-0054] Vet, L. E. M. , Datema, A. , Janssen, A. , & Snellen, H. (1994). Clutch size in a larval‐pupal endoparasitoid: Consequences for fitness. Journal of Animal Ecology, 63, 807–815.

[ece32826-bib-0055] Vet, L. E. M. , & Dicke, M. (1992). Ecology of infochemical use by natural enemies in a tritrophic context. Annual Review of Entomology, 37, 141–172.

[ece32826-bib-0056] Via, S. (1999). Reproductive isolation between sympatric races of pea aphids. I. Gene flow restriction and habitat choice. Evolution, 53, 1446–1457. 10.1111/j.1558-5646.1999.tb05409.x 28565574

[ece32826-bib-0057] Zaviezo, T. , & Mills, N. (2000). Factors influencing the evolution of clutch size in a gregarious insect parasitoid. Journal of Animal Ecology, 69, 1047–1057.

