# Supplementary material for: Natal origin affects host preference and larval performance relationships in a tritrophic system
Source: Ecol Evol. 2017 Feb 26;7(7):2079–90. doi: 10.1002/ece3.2826 (PMC5383469; doi:10.1002/ece3.2826)
Supplement: Supplementary file 1 [file ECE3-7-2079-s001.docx]

**Natal origin affects host preference and larval performance relationships in a tritrophic system**

Online Supporting Information

**Thomas A. Verschut^a*^, Laima Blažytė-Čereškienė^b^, Violeta Apšegaitė^b^, Raimondas Mozūraitis^a, b^ and Peter A. Hambäck^a^**

^a^Department of Ecology, Environment and Plant Sciences, Stockholm University, 106 91 Stockholm, Sweden

^b^Laboratory of Chemical and Behavioural Ecology, Institute of Ecology, Nature Research Centre, Akademijos st. 2, LT-08412 Vilnius, Lithuania

**Corresponding author:** thomas.verschut@su.se

**Online supporting information**

**Fig. S1.** Behavioral responses of natal *Potentilla* (n=23 and n=22 respectively) and natal *Lysimachia* (n=24 and n=24) beetles to odors from the undamaged *Potentilla palustris* and *Lysimachia thyrsiflora*. The x-axis shows the mean number of visits per arm with the standard error of the mean. The control treatments (white bars) consisted of humidified air. All other odor treatments in combination with the natal origin of the beetles are given on the left side of the bars.

**Figure S2.** The correlation between mummy length and hind tibia length of females (circles) and males (squares) for parasitoids hatching from *Potentilla palustris* (grey markers and dashed line) and *Lysimachia thyrsiflora* (black markers and solid line).

| **Table S1.** Likelihood ratio tests (*X^2^)* for the behavioral response of *Galerucella sagittariae* females from both natal origins to odors emitted by current and natal host plants of either species (damaged or undamaged). | | | | |
| --- | --- | --- | --- | --- |
| **Experiment** | **Factors** | ***X^2^*** | ***Df*** | **P** |
| One-choice | Natal origin | 1.205 | 1 | 0.272 |
|  | Damage | 13.171 | 1 | <0.001 |
|  | Current host plant | 0.225 | 1 | 0.635 |
|  | Natal origin *x* Current host plant | 0.944 | 1 | 0.331 |
|  | Damage *x* Current host plant | 0.357 | 1 | 0.550 |
| Two-choice | Natal origin | 1.559 | 1 | 0.212 |

| **Table S2.** Overview of the EAD active compounds in the headspace of the damaged host plants. The retention index (RI) is based on a DB-Wax column. FID gives the averaged absolute peak area ± standard error of the mean as detected by Flame Ionization Detector. EAD gives the averaged absolute peak heights ± standard error of the mean of the antennal response to each compound. γ-Caprolactone is a tentative identification of the compound. Values are based on *n=*5 for *Potentilla palustris* and *n=*4 for *Lysimachia thyrsiflora.* | | | | | | | |
| --- | --- | --- | --- | --- | --- | --- | --- |
|  |  |  |  | ***Potentilla palustris*** | | ***Lysimachia thyrsiflora*** | |
| **RI** | **Compound** | **Class** | **CAS No** | **FID** | **EAD** | **FID** | **EAD** |
| 1063 | Hexanal | Aldehyde | 66-25-1 | 16233 ± 2904 | 25.9 ± 8.3 | 12564 ± 3688 | 21.2 ± 8.7 |
| 1130 | (*Z*)-3-Hexenal | Aldehyde | 6789-80-6 | 99436 ± 15590 | 62.3 ± 14.5 | 262901 ± 102827 | 84.1 ± 28.5 |
| 1206 | (*E*)-2-Hexenal | Aldehyde | 6728-26-3 | 32366 ± 2430 | 44.9 ± 9.7 | 57152 ± 16552 | 65.4 ± 14.7 |
| 1250 | (*E*)-β-ocimene | Monoterpene | 13877-91-3 | 1212604 ± 180920 | 22.1 ± 8.3 | 5836 ± 2044 | 28.2 ± 0 |
| 1314 | (Z)-3-Hexenyl acetate | Ester | 3681-71-8 | 153096 ± 16565 | 54.7 ± 7.5 | 11794 ± 3966 | 33.2 ± 7.6 |
| 1356 | Hexanol | Alcohol | 111-27-3 | 14652 ± 2081 | 29.9 ± 5.7 | 2081 ± 457 | 17.7 ± 6.5 |
| 1384 | (*Z*)-3-Hexanol | Alcohol | 928-96-1 | 172781 ± 19724 | 78.0 ± 17.3 | 159816 ± 67966 | 81.6 ± 25.7 |
| 1405 | (*E*)-2-Hexenol | Alcohol | 928-95-0 | 3038 ± 244 | 40.4 ± 3.8 | 6630 ± 2181 | 59.4 ± 21.5 |
| 1415 | Hexyl-2-metylbutanoate | Ester | 10032-15-2 | 3710 ± 791 | 9.9 ± 4.1 | - | - |
| 1461 | (*E*)-3-Hexenyl butyrate | Ester | 53398-84-8 | 58993 ± 5923 | 53.4 ± 12.6 | 3418 ± 682 | 40.0 ± 14.2 |
| 1485 | (*E*)-Myroxide | Oxygenated Monoterpene | 94607-47-3 | 171456 ± 15873 | 12.7+5.4 | 2845 ± 1288 | - |
| 1688 | γ-Caprolactone | Lactone | 695-06-7 | 33843 ± 8894 | 72.9 ± 19.0 | 1485 ± 608 | 24.0 ± 17.1 |
| 2038 | trans-Nerolidol | Oxygenated Sesquiterpene | 40716-66-3 | 4060 ± 775 | 24.5 ± 8.9 | 6716 ± 1002 | 18.8 ± 3.0 |

| **Table S3.** Summary of likelihood ratio tests (*X^2^)* for the olfactory preference of *Asecodes lucens* in Y-tube olfactometer experiments. The odour source refers to either *G. sagittariae* larvae or the host plants emitting the odour cues. The current host plant refers to the host plant species of which the odour was emitted during the experiment. The natal origin corresponds to the combination of *G. sagittariae* larvae and host plant species from which the *A. lucens* females derived. | | | | |
| --- | --- | --- | --- | --- |
| **Experiment** | **Factors** | ***X^2^*** | ***Df*** | **P** |
| One-choice | Odour source | 7.149 | 1 | 0.007 |
|  | Current host plant | 0.403 | 1 | 0.525 |
|  | Odour source *x* Current host plant | 0.149 | 1 | 0.699 |
| Two-choice | Natal origin | 14.506 | 1 | <0.0001 |

**Appendix S1**

*Headspace collection of plant volatiles and chemical analysis*

To quantify differences in odor profile between plants, we collected organic compounds (VOCs) from the headspace of undamaged and feeding damaged host plants through dynamic aeration in a push-pull system. Feeding damage was obtained as described in the previous section and volatile collections were performed after beetles were removed. Shoots of approximately the same volume were placed in glass containers (⌀ 6.0 cm x 20 cm) with a guillotine base with a small opening (⌀ 8 mm) for plant stem and air delivery (Millar and Sims 1998, Tholl et al. 2006). Humidified and purified air was pushed into the system at 150 mL/min and transported the VOCs towards the top of the glass container. Here, a second diaphragm vacuum pump (NMP 830, KNF Neuberger GmbH, Germany) pulled air at 130 mL/min through a glass tube collection trap (⌀ 5 mm x 6.0 cm) filled with 50 mg of Tenax TA absorbent (60/80 mesh; Sigma-Aldrich AB, Sweden). The difference in airflow ensured that the system was continuously purged through the hole of the guillotine base and no contaminated air would enter the system. The odor collection traps were extracted every four hours with 250 µl of redistilled diethyl ether until 20 h of headspace collection was reached. The extracts from the same plant were combined with an addition of 20 ng of pentadecane as internal standard and concentrated to 10 µl. Prior to analysis the samples were concentrated under a gentle flow of nitrogen and a maximum of 1 µL was injected in a HP 6890N gas chromatograph equipped with a DB-Wax capillary column (30 m x 0.25 mm x 0.25 µm; Agilent Technologies, USA) coupled to a HP 5973 mass spectrometer (Agilent Technologies Inc, USA). The injector and the detector temperatures were set isothermal at 230 °C and 240 °C respectively with the oven temperature programmed from 40 °C for 3 min, then 4 °C min^-1^ to 200 °C, then 10 °C min^-1^ to 240 °C and afterwards held isothermal at 240 °C for 15 min. Helium was used as the carrier gas with a constant flow through the column at 0.9 mL/min^-1^. Electron ionization mass spectra were determined at 70 eV with the ion source at 200 **°**C. After sampling we removed all leaves from the stem and scanned the total leaf surface area and the damaged surface area using the software ImageJ v.1.48.

# *Coupled gas chromatography – electroantenographic detection*

# In order to determine which VOCs elicit antennal responses in *G. sagittariae* we performed coupled gas chromatographic–electroantennographic detection (GC-EAD). VOCs collections for the GC-EAD were made with 10 adult beetles feeding on the plant. Collections from the same plant species were combined reaching total collection time of 160 h, and concentrated to 10 µL with 200 ng of pentadecane as internal standard. A total of 1 µL of the concentrated extract was injected for GC-EAD recordings, and the EAG responses were measured on randomly selected reproductive mature females from both natal origins as follows. The head was detached and a silver wire grounded glass capillary electrode filled with 0.9% NaCl saline solution (Ilsanta, Lithuania) was inserted into the beetle’s head. Thereafter, the distal end of the antennae was connected to a recording electrode and the antennal signal was amplified 10 times, converted to a digital signal by a high input impedance DC amplifier interface (IDAC-4, Syntech, Germany) and recorded simultaneously with the FID signal (GC-EAD V.4.4; Syntech, Germany). Prior to each recording the prepared antennae was held in a humidified air-stream at 0.5 m/s and the responses to direct stimulation of 10 μl hexanol solution in hexane (1mg/mL) was tested. The GC-EAD recordings were made on a Clarus 500 gas chromatograph (PerkinElmer, USA) equipped with a DB-Wax capillary column (30 m x 0.25 mm x 0.25 µm; Agilent Technologies, USA) in which a 1:1 effluent splitter allowed a simultaneous flame ionization detection (FID) and EAG detection of the separated volatile compounds with hydrogen as a carrier gas (1.5 mL/min). The injector and the detector temperatures programmed from 40 °C for 2 min, then 5 °C min^-1^ to 200 °C, then 10 °C min^-1^ to 240 °C and held at 240 °C for 12 min.

In total five GC-EAD recordings were averaged to obtain consistent antennal responses, and the EAG active compounds were identified by firstly comparing their mass spectra with those in the NIST electronic MS library (V2.0; National Institute of Standard and Technology, USA), then to published retention time values and finally, with authentic standards using the MSD Productivity ChemStation software version E.02.01.1177 (Agilent Technologies Inc., USA). The relative amounts of the compounds injected were determined as areas under chromatographic peaks.

*References*

Millar, J. G., and J. J. Sims. 1998. Preparation, cleanup, and preliminary fractionation of extracts.*in* M. J.G. and K. F. Haynes, editors. Methods in Chemical Ecology Vol. 1 - Chemical Methods. Kluwer Academic Publishers, Boston, USA.

Tholl, D., W. Boland, A. Hansel, F. Loreto, U. S. R. Röse, and J. P. Schnitzler. 2006. Practical approaches to plant volatile analysis. The Plant Journal **45**:540-560.
